# A Stochastic Model for Microtubule Motors Describes the *In Vivo* Cytoplasmic Transport of Human Adenovirus

**DOI:** 10.1371/journal.pcbi.1000623

**Published:** 2009-12-24

**Authors:** Mattia Gazzola, Christoph J. Burckhardt, Basil Bayati, Martin Engelke, Urs F. Greber, Petros Koumoutsakos

**Affiliations:** 1 Chair of Computational Science, ETH Zurich, Zurich, Switzerland; 2 Institute of Zoology, University of Zurich, Zurich, Switzerland; University of Washington, United States of America

## Abstract

Cytoplasmic transport of organelles, nucleic acids and proteins on microtubules is usually bidirectional with dynein and kinesin motors mediating the delivery of cargoes in the cytoplasm. Here we combine live cell microscopy, single virus tracking and trajectory segmentation to systematically identify the parameters of a stochastic computational model of cargo transport by molecular motors on microtubules. The model parameters are identified using an evolutionary optimization algorithm to minimize the Kullback-Leibler divergence between the *in silico* and the *in vivo* run length and velocity distributions of the viruses on microtubules. The present stochastic model suggests that bidirectional transport of human adenoviruses can be explained without explicit motor coordination. The model enables the prediction of the number of motors active on the viral cargo during microtubule-dependent motions as well as the number of motor binding sites, with the protein hexon as the binding site for the motors.

## Author Summary

Molecular motors, due to their transportation function, are essential to the cell, but they are often hijacked by viruses to reach their replication site. Imaging of virus trajectories provides information about the patterns of virus transport in the cytoplasm, leading to improved understanding of the underlying mechanisms. In turn improved understanding may suggest actions that can be taken to interfere with the transport of pathogens in the cell. In this work we use *in vivo* imaging of virus trajectories to develop a computational model of virus transport in the cell. The model parameters are identified by an optimization procedure to minimize the discrepancy between *in vivo* and *in silico* trajectories. The model explains the *in vivo* trajectories as the result of a stochastic interaction between motors. Furthermore it enables predictions on the number of motors and binding sites on pathogens, quantities that are difficult to obtain experimentally. Beyond the understanding of mechanisms involved in pathogen transport, the present paper introduces a systematic parameter identification algorithm for stochastic models using *in vivo* imaging. The discrete and noisy characteristics of biological systems have led to increased attention in stochastic models and this work provides a methodology for their systematic development.

## Introduction

The function of eukaryotic cells relies on the transport of macromolecules and organelles throughout the cytoplasm. Pathogenic viruses can exploit a cell's cytoplasmic transport mechanisms [1],[2] in order to reach their site of replication. Cytoplasmic transport involves three types of molecular motors. Kinesin and dynein motors use microtubule tracks to move cargo throughout the cytoplasm, while myosin motors interact with actin filaments to move their cargoes [3],[4]. Microtubule based transport is usually bidirectional and its mechanism can be explained by the exclusive binding of dynein and kinesin motors to the cargo, motor cooperation and regulation, or a stochastic tug-of-war [5]–[8]. Exclusive binding of motors has not been reported in cells, while in systems with cooperating motors, additional factors such as on/off switches or coordinators between motors have been postulated for bidirectional transport of large cargo, such as vesicles [7]. The mechanism of bidirectional motor transport by non-coordinated motors of opposite polarity has been the basis of tug-of-war models [7],[9].

**Figure 1:**
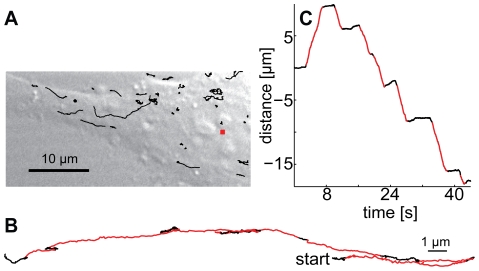
Imaging, tracking and trajectory segmentations of single adenoviruses. (A) HeLa cells were infected with fluorescent adenovirus type 2 for 30 min, and imaged by spinning disc confocal fluorescence microscopy [42]. Virus tracks (black lines) recorded by a single particle tracking algorithm [10] using the nucleus (red square) as a reference point are displayed over a phase contrast image of the infected cell. (B) Two-dimensional projection of a single virus trajectory with directed motion segments in red. (C) Distance travelled along the trajectory shown in Fig. 1B plotted as a function of time. Reduction to 1D is justified, since in cultured cells microtubules are largely flat and straight over distances of many micrometers [43]. Negative/positive values indicate displacements towards the cell centre/periphery.

In this work we propose a stochastic model for motor transport on microtubules and we systematically identify its parameters using virus trajectories obtained by *in vivo* imaging (Fig. 1). Trajectories are obtained by live cell microscopy of fluorescently labelled human adenovirus type 2 (Ad2) using confocal microscopy. Motility information extracted through single virus tracking [10], and trajectory segmentation [11] are implemented in order to study the properties of virus transport by employing a systems identification process [12] for a stochastic model of cargo transport on microtubules.

### A Stochastic Model for Cargo Transport

The small number of motor proteins involved in microtubule transport implies a system where the fluctuations in the behavior of motors and the randomness of molecular reactions are essential characteristics [13] suggesting a stochastic modeling of the governing processes. Here we propose a stochastic representation of the main events involved in motor transport, namely stepping along microtubules and binding and unbinding of molecular motors to the cargo.

The proposed model has six parameters, namely the binding, unbinding and stepping rates of plus-end and minus-end motors (herein presumed to be dynein and kinesin, respectively). The step sizes of the motors were set to −8/+8 nm for dynein/kinesin as suggested by the results of single molecule experiments [14],[15]. We note that we do not impose any geometrical information on the motors and their binding sites on the virus capsid. The motor protein binding sites on the adenovirus capsid are not known even though a recent cryo-EM image of the structure of the human adenovirus type 2 temperature sensitive mutant revealed the organization of the surface of the virus capsid [16].

The six model parameters are inferred through a system identification process using the velocity and displacement distributions of segmented trajectories as the cost function of our optimization. An evolutionary algorithm, capable of handling noisy cost functions, is used to obtain the rates that minimize the distance between the velocity and displacement distributions of the *in silico* and *in vivo* trajectories.

**Figure 2:**
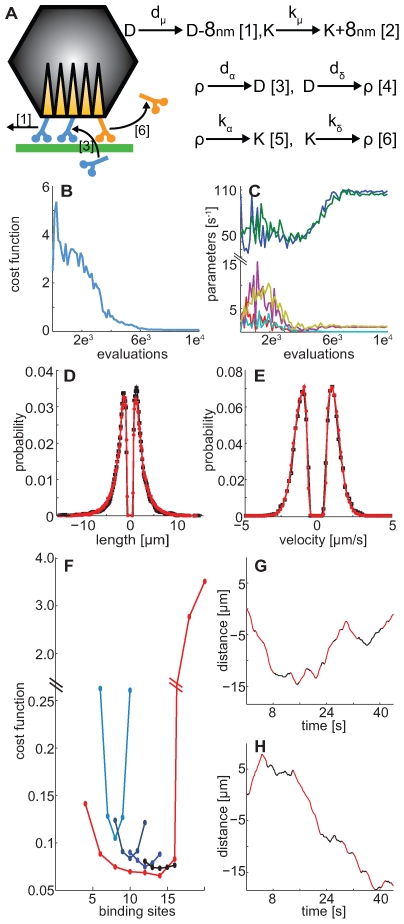
A stochastic model for microtubule-dependent movements of human adenovirus. The stochastic model reproduces directed motion length and velocity distributions of human adenovirus, and predicts the optimal number of either common or separate binding sites for dynein and kinesin motors on the capsid. (A) Dynein (D, blue) and kinesin (K, orange) bind to, and unbind from the capsid and transport it along a microtubule (green). Equations [1]–[6] describe the dynamics of the model: [3]/[4] dynein binding/unbinding and decrease/increase of the number (ρ) of the available motor binding sites on the virus capsid with d_α_/d_δ_ binding/unbinding rates, [5]/[6] kinesin binding/unbinding and decrease/increase of the number(ρ) of the available motor binding sites on the virus capsid with k_α/_k_δ_ binding/unbinding rates, and [1]/[2] dynein/kinesin motor stepping with d_μ_/k_μ_ stepping rates. Cost function (B) and parameter values (C) (blue = d_μ_, green = k_μ_, red = d_α_, cyan = k_α_, yellow = d_δ_, magenta = k_δ_) versus number of evaluations during the optimization of the 14 common binding sites model. Probability distribution of directed motion length (D) and velocity (E) for the *in vivo* and *in silico* (black/red) trajectories. (F) Plot of the cost function versus the number of motor binding sites for the common (red) and the separate binding sites model (blue, grey, dark blue, black colours). The separate binding sites have a total number of 8 (blue), 10 (grey), 12 (dark blue) and 14 (black) binding sites for dynein plus kinesin motors. The central dot in each curve represents 50% dynein and 50% kinesin occupancy (e.g. black curve: 7+7). The remaining dots denote permutations with decreasing/increasing dynein binding sites (e.g. 6 dynein + 8 kinesin on the right and 8 dynein +6 kinesin on the left of the central dot of the black curve). (G, H) examples of segmented *in silico* 1D trajectories for the 14 common (G) or 7+7 separate (H) binding sites models. The distance (µm) traveled along the 1D microtubule is plotted against the time in seconds, and the directed motions are depicted in red.

The velocity distribution in virus trajectories has led to several suggestions regarding the cooperation or lack thereof between molecular motors. High velocities, in the order of a few microns per second, were observed for intracellular viruses (Fig. 2E) [17]. Similar high speeds have been observed for vesicles moving along microtubules such as peroxisomes [18] and endosomes [19]. These velocities are above the maximum velocities measured for single motors without load (3 µm/s for dynein, [14]; 0.4 µm/s for kinesin-1, [20]; 3 µm/s for kinesin-1, [21]; 0.8 µm/s for kinesin-1, [22]; 0.8 µm/s kinesin-1 and 0.5 µm/s kinesin-2, [23] in *in vitro* experiments. It has also been reported for drosophila lipid droplets, that multiple processive motors do not move cargoes faster [24]. It is likely that yet unknown mechanisms account for the high velocities measured in *in vivo* biological systems. These mechanisms may involve motors which are able to increase their velocities up to few microns per second or motors are able to act additively to achieve higher speeds. Both assumptions have not been experimentally validated or discarded in *in vivo* experiments. Additive behaviour of motors is an underlying assumption in our model (Fig. 2A). The additive behaviour is inherent to the Stochastic Simulation Algorithm [25] used herein to simulate the model, since the time step to the next event depends on the total propensity (numbers and event rates). The proposed stochastic model does not impose any explicit coordination between motor proteins, e.g. a switching mechanism that selects a certain motor protein type to be active.

We emphasize that our model does not aim at a mechanistic description at the motor level. Forces are known to affect motor properties, but it is not clear how they are distributed among multiple motors [26]. Furthermore while it is possible to obtain data relating forces for certain motors *in vitro*, there is no such data for *in vivo* experiments. In the present model the forces between molecular motors and cargo are implicitly taken into account through the binding/unbinding/stepping rates of the stochastic models.

## Results

The simulation of the stochastic model produces cargo trajectories that depend on the parameter settings. The model contains no a-priori assumptions on the existence of either a tug-of-war or coordination between molecular motors. In turn, the model parameters are systematically identified with a derandomized evolution strategy that minimizes the difference between the length and velocity distributions of directed motions (fast microtubule dependent runs [11]) produced by the model and those of fluorescently labelled human adenovirus type 2 (Ad2) as measured by confocal microscopy at 25 Hz temporal resolution. The two-dimensional virus trajectories are extracted by a single particle tracking algorithm [10] (Fig. 1A, B). Directed motions along microtubules are classified by trajectory segmentation [11] and the distance travelled along the microtubule is determined as a function of time (1D trajectory shown in Fig. 1C). The same analysis is applied to trajectories obtained by the simulation of our model using the Stochastic Simulation Algorithm (SSA) [27]. These trajectories are also subsequently segmented to classify directed motions [11]. In turn an optimization algorithm is employed to identify the parameters of the stochastic model [28].

**Figure 3:**
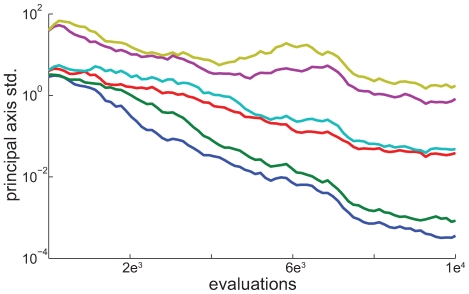
Convergence of the standard deviation of the principal axes. Convergence is shown for the six axes of the distribution which CMA-ES samples from. The evolution of the standard deviations during the optimization procedure is shown for the non-competing binding sites model with 14 receptors.

Here the six model parameters (binding, unbinding and stepping for both kinesin and dynein, Fig. 2A) were identified by minimizing the Symmetric Kullback-Leibler divergence between the *in silico* and *in vivo* length and velocity distributions using an Evolution Strategy with Covariance Matrix Adaptation (CMA-ES) [29] (Fig. 2B,C). The proposed de-randomized optimization algorithm is an essential aspect of our method. CMA-ES samples the six-dimensional multivariate normal distribution involving the parameters of this problem at each iteration and it encodes relations between the parameters of the model and the objective that is being optimized without requiring explicitly the gradients of the cost function [29]. The CMA-ES is a method capable of optimizing noisy cost functions (such as those from the present stochastic model) and its efficiency, reliability and robustness were demonstrated over a number of benchmark problems [30],[31]. The CMA-ES is particularly suitable to this optimization problem as it is know to perform best [29] in problems that are low dimensional (here six parameters), inherently noisy (here a stochastic model), multimodal and computationally expensive (for each parameter set thousands of trajectories are generated and segmented to collect reliable statistics). The algorithm identifies an optimal set of parameters and at the same time provides a sensitivity analysis of the model. The standard deviations of the six principal axes are shown to converge (Fig. 3), thus yielding a minimum (Text S1).

After the convergence of the optimization process (Fig. 3) we found that the directed motion length and velocity distributions of the *in silico* trajectories, under the optimal set of parameters, matched with high accuracy the experimental data (Fig. 2D, E).

The maximum number of motors attached to the viral cargo is limited by the number of binding sites on the virus. The present model enables predictions on the number of motor binding sites on the viral capsid, a quantity that is difficult to determine experimentally but important for understanding the mechanisms of transport. We first estimated the number (between 2 and 20) of motor binding sites on the virus by an optimization procedure (Fig. 2F). In models with 6–16 binding sites, the cost function values were almost constant around the minimum value obtained for 14 binding sites (Text S1). For less than 6 motor binding sites, the optimization process did not converge to the experimentally observed directed motion length and velocity distributions. Above 16 binding sites, an unbalanced configuration of motors was feasible only at low binding and unbinding rates, and yielded largely unidirectional trajectories due to infrequent motor binding to the virus. We concluded that 14 common binding sites for dynein and kinesin correspond optimally to the experimental data.

Since it is not possible to differentiate between common and separate binding sites, we additionally investigated the possibility that the experimental data support separate binding sites for the different motors. We optimized a model where dynein and kinesin have distinct binding sites, namely 4+4, 5+5, 6+6, 7+7 binding sites, and various permutations thereof (Fig. 2F, Text S1), and found that an equal number of motor binding sites was optimal in all cases. This is consistent with the observation that center and periphery directed length and velocity distributions were almost symmetric (Fig. 2D, E). We note that the optimal number of binding sites, i.e., 14, is the same for the models with common and separate binding sites (Fig. 2F, black curve).

**Figure 4:**
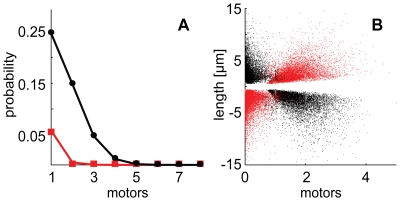
A low number of dynein and kinesin motors mediate directed motions of adenovirus. (A) *In silico* probabilities of the number of bound dynein (black) and kinesin (red) motors during periphery directed motions. Similar results were observed for center directed motions with dynein prevalence. Phase diagrams of the length (B) of directed motions versus the average number of bound dynein (black) and kinesin (red). Positive (negative) lengths correspond to periphery (center) directed motions. The results were obtained with the optimized model with 14 common binding sites.

Molecular motors carrying cargo on microtubules operate as individuals or as an ensemble. We found that, on average, during virus directed motions, 1.56±0.56 dynein or kinesin (for minus-end and plus-end directed motions, respectively) motors, and 0.15±0.22 motors of opposite polarity were bound to the virus (Fig. 4A). The probability of binding more than four motors to one virus particle was below 10^−3^, and most often only one type of motor was bound (Fig. 4A, B). These data are in agreement with low number of motors estimated on vesicular cargo in squid axoplasm by cryo-EM [7]. For other organelles, the estimates for motor numbers range from a few to dozens based on immunological detections in chemically fixed cells.

In order to quantify the correlation between the number of bound motors and the directed motion length, the Sample Pearson Product Moment correlation coefficient (with a range of 0 to 1, where 1 is maximal correlation) between motor numbers and directed motion length was computed to be 0.51 for dynein and 0.49 for kinesin for minus-end and plus-end directed motions, respectively. This implies a weak correlation between the number of bound motors and the directed motion length, showing that long runs do not necessarily require many motors, as two or three already account for lengths in the order of micrometers (Fig. 4B). This result is consistent with the recent *in vitro* observation that two motors are sufficient to move a cargo over several micrometers [32].

The velocities, derived from optimized stepping rates, for single dynein and kinesin motors were 866 nm/s and 833 nm/s, respectively, consistent with values reported for dynein and conventional kinesin-1 or kinesin-2 [21]–[23],[33]. Although kinesins are currently not known to be involved in cytoplasmic transport of adenovirus [1], the model makes a clear prediction for a plus end directed motor in cytoplasmic transport of adenovirus.

Our findings indicate that microtubule-based motility of adenovirus requires a low number of bound motors compared to the number of binding sites on the capsid. This allows configurations where only one motor type is bound, and thereby produce directed motions. Low numbers of motors allow fast switches between directions and therefore, bidirectional motion. Importantly, the binding and unbinding rates were much smaller than the stepping rates, which is key for directed motion runs (Fig. 2C). Small perturbations of binding and unbinding rates greatly affect the model dynamics (Text S1). The susceptibility of motor based cargo transport to these parameters has been reported in other theoretical studies [26] and hints to a possible mechanism to regulate the run length of the motors [32].

**Figure 5:**
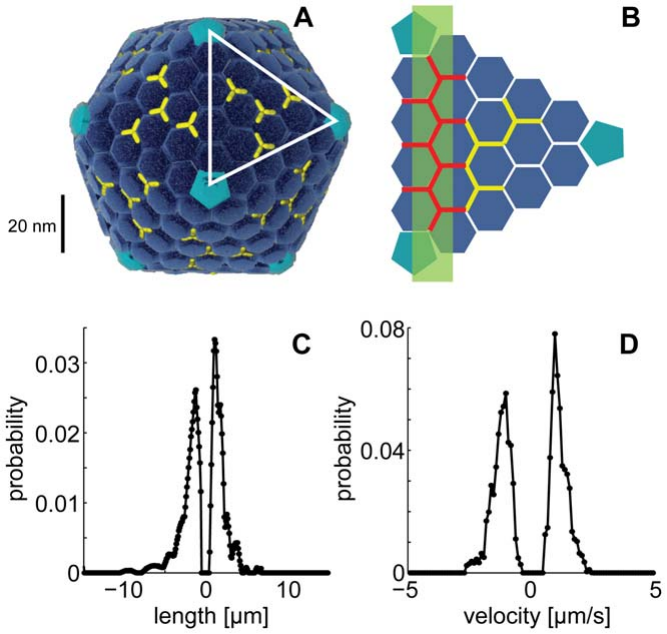
Hexon not protein IX is the predicted to be motor binding site on the adenovirus capsid. (A) Schematic model of the icosahedral adenovirus capsid with the major capsid protein hexon (blue hexagonal structures representing trimers), and pIX (yellow lines representing trimers). An icosahedral facette is enlarged in (B), where hexon-hexon trimer interfaces are depicted in red and in light green the microtubule orientation that maximizes the overlap with one facet. Note that the fourth trimer of pIX is covered by the red lines depicting hexon-hexon interfaces. (C, D) Directed motion length and velocity distributions for protein IX deficient adenovirus recorded in HeLa cells 30 to 90 min post infection.

The present results enabled an assessment on the virus binding sites for motor proteins. The outer surface of adenovirus particles is composed of five polypeptides, three of which are still present on cytosolic viruses that have undergone stepwise disassembly [34]. Cytosolic particles contain the major protein hexon, a large fraction of the pentameric penton base at the icosahedral vertex, and protein IX (pIX), which stabilizes hexon. By considering the size (90 nm in diameter) and icosahedral geometry of the virus and the cylindrical microtubule (25 nm in diameter), we can postulate that the maximum number of microtubule motor-capsid interactions occurs along the edge of a capsid facet, in this case on hexon (Fig. 5A, B). This arrangement implies that 9 hexon trimers are aligned with the microtubule, giving a maximum of 27 hexon binding sites for the motors. This is above the value of 14 binding sites predicted from the simulations. If we assume, however, that the motor protein binding sites are located at the interface of two trimeric hexons, one microtubule filament could cover 1–15 sites (Fig. 5B, red lines), which is within the predicted range of 6–16. In addition to hexon, 6 to 8 binding sites were available for pIX, and less than 5 for penton base which detaches to a significant extent from the incoming virions before reaching the cytosol [34]. We analyzed trajectories of pIX-deficient adenoviruses to distinguish between hexon and pIX binding sites for motor proteins [35]. The directed motion length and velocity distributions of pIX-deleted adenovirus were similar to those from wild-type viruses without significant deviations or asymmetries, indicating that pIX may not provide a binding site for microtubule dependent motors during cytoplasmic transport (Fig. 5C, D). Therefore, we predict that hexon harbours the binding sites for dynein and kinesin motors.

## Discussion

In this study, we use *in vivo* imaging to identify a stochastic model of cargo transport by molecular motors on microtubules. The model parameters were systematically identified using live imaging data of virus trajectories and a de-randomized optimization algorithm to minimize the Kullback-Leibler divergence between the length and velocity distributions of adenovirus directed motions on microtubules with the *in silico* trajectories produced by the model. The model accounts for directed motions at µm/s speeds, processive stepping over hundreds of nanometres, and periods of stationary behaviour. The results show that the stochastic model can result in bidirectional support without an explicit coordination mechanism.

In our work kinetic rates of a stochastic model are determined via an evolutionary optimization approach using experimental data. The identified model enables a number of predictions. First, it shows that one to four motors are active on virus particles during microtubule-dependent motions, although the number of motor binding sites is estimated to be 6–16. The observation that the cost function value is constant within this range suggests that the virus may align with the microtubule in different orientations (Fig. 5B) and still preserve its motility. This range is consistent with the maximum of 15 hexon trimer-trimer interfaces along the edge of a capsid facet. The low number of motors involved in directed motions supports an emerging concept from wet lab experiments and *in silico* simulations, that key events of cell functions are in many cases executed by only a few polypeptides [36].

Second, if equal numbers of opposite motors are attached, the cargo oscillates and eventually stops, or remains confined to small areas. This may be an important mechanism for fine-tuning the subcellular velocity to achieve localized delivery of the cargo. We anticipate that viral transport is tuned by the binding and unbinding rates of motors to microtubules or the cargo, rather than by additional regulatory factors. Such tuning could be cell-type specific [17], and could control the number of engaged motors and motor configuration, and also provide specific segregation or guidance cues for traffic. In support of this, it has been suggested that the microtubule binding protein Tau can fine-tune the distance that the cargo travels by reducing microtubule binding of kinesin in distal parts of neuronal axons [7],[37]. In addition, motor properties can be tuned by post-translational modifications, such as phosphorylation of dynein or kinesin binding partners, which could affect their enzymatic functions and hence their stepping rates [7].

We close by noting that besides the results on motor transport on microtubules, the algorithm taken here is in line with reverse engineering and systems identification approaches [28], [38]–[40] which are gaining significance as discovery and model validation tools in systems biology. The CMA-ES algorithm is capable of handling noisy and multimodal cost functions that are inherently associated with stochastic models. The CMA-ES optimization algorithm along with image analysis of *in vivo* systems can be a robust process to help identify parameters of stochastic models employed in several areas of systems biology.

## Materials and Methods

HeLa cells were grown to 30% confluency on 18 mm glass cover slips (Menzel Glaser) and kept in Hanks buffered salt solution containing 0.5% BSA (Sigma) and 1 mg/ml ascorbic acid (Sigma). Adenovirus serotype 2 and protein IX deficient adenoviruses were grown, isolated, and labeled with atto565 (Atto-tec, Germany) as described by Nakano and Greber in [41] and Suomalainen et al. in [17].

HeLa cells were infected with Ad-atto565 and imaged between 30 and 90 minutes post infection at 25 Hz. Flat regions of the cell were chosen for imaging in order to minimize the cytoplasmic volume above the imaging plane. The center of the cell was detected by differential interference contrast imaging to assign directionality to the virus motions. Images were recorded using a spinning disc confocal microscope (Olympus IX81) fitted with an UplanApo100x objective of N.A. 1.35 on a back-illuminated monochrome Cascade 512 EM-CCD camera (Photometrics) containing a 512×512 pixel chip (with 16×16 micrometer large pixels).

For the computational methods see Text S1.

## Supporting Information

Text S1 Supplementary information includes details on the computational methods used. In particular it describes the trajectory segmentation process, the models studied, the stochastic simulation algorithm and the definition of the cost function used in the optimization procedure.
(1.05 MB PDF)
