# Supplementary material for: A Stochastic Model for Microtubule Motors Describes the In Vivo Cytoplasmic Transport of Human Adenovirus
Source: PLoS Comput Biol. 2009 Dec 24;5(12):e1000623. doi: 10.1371/journal.pcbi.1000623 (PMC2789326; doi:10.1371/journal.pcbi.1000623)
Supplement: Text S1 — Supplementary information includes details on the computational methods used. In particular it describes the trajectory segmentation process, the models studied, the stochastic simulation algorithm and the definition of the cost function used in the optimization procedure. (1.05 MB PDF) [file pcbi.1000623.s001.pdf]

A stochastic model for microtubule motors describes the *in vivo* cytoplasmic transport of human adenovirus

Mattia Gazzola[1], Christoph J. Burckhardt[2], Basil Bayati[1], Martin Engelke[2], Urs F. Greber[2*)], Petros Koumoutsakos[1*)]

[1]Chair of Computational Science, ETH Zurich, CH-8092, Switzerland

[2] Institute of Zoology, University of Zurich, CH-8057, Switzerland

[*)] corresponding authors: ufgreber@zool.uzh.ch and petros@ethz.ch

Supplementary Information

Trajectory Transformation from 2D to 1D. Each biological trajectory is converted to 1D by connecting time-consecutive points, calculating the run length along the track, and then plotting it against the time (i.e. the frame number). The direction of the run is determined by examining the position of the particle relative to the cell center, where a minus or a plus sign is assigned if the particle moves towards the cell center or the periphery, respectively. The set of biological trajectories translated to 1D is used for two purposes. First, it represents the training set for the SVM to segment the 1D *in silico* trajectories (see section Trajectory Segmentation by Supervised Support Vector Machine). In this case the SVM was trained with 60 randomly selected 1D biological trajectories, where the directed motion labels were retained from the 2D data set. Second, it is used to compute directed motions length and velocity probability distributions (PDFs) (see section Optimization of Parameters).

Trajectory Segmentation by Supervised Support Vector Machine The images of infected cells were processed in order to ascertain directed motion patterns. A single particle tracking algorithm [1] was applied to the images, and consequently, 2D trajectories of virus particles were obtained. Following that, the trajectories were segmented using a Support Vector Machine (SVM) algorithm [2,3] to extract directed motions. The segmentation process involved a training and a prediction stage. During the training the SVM is presented with a set of β feature vectors of dimension n (number of features) that had specified class labels (e.g. pattern or not). In the case of 2D trajectory segmentation, positive and negative examples of directed motion patterns were manually selected. The SVM identifies the maximum-margin separating hyperplane between vectors belonging to different classes. After training, the SVM represents a model that

assigns a class label to a given feature vector (prediction stage).

Given the training feature vectors $\mathbf{x}_i \in \Re^n$, $i = 1,...,\beta$ and the class label vector $\mathbf{y} \in \{1,-1\}^\beta$, SVM determines the maximum-margin separating hyperplane ($\mathbf{w}$,b) by solving the following simplex optimization problem:

$$\min_{\mathbf{w},b}: \quad f(\mathbf{w},\mathbf{s}) = \frac{1}{2}\mathbf{w}^T\mathbf{w} + C\sum_{i=1}^{\beta} s_i \qquad (1)$$

$$\text{s.t.:} \quad (\mathbf{w}^T\mathbf{x}_i + b)y_i \geq 1 - s_i \qquad i = 1,...,\beta \qquad (2)$$

$$s_i \geq 0 \qquad i = 1,...,\beta \qquad (3)$$

where C>0 is the penalization parameter for the error term (Eq. 1) and s_i are the so-called slack variables. Non-linear classifiers can be obtained replacing every dot product by non-linear kernel function. The kernel function $K(\mathbf{x}_{i,}\mathbf{x}_j)$ determines how feature vectors $\mathbf{x}_i$ are mapped into a higher dimensional space, and the SVM finds in this space the hyperplane that separates the data so as to minimize the error. The radial basis function kernel, $K(\mathbf{x}_i,\mathbf{x}_j) = e^{-\gamma\|\mathbf{x}_i - \mathbf{x}_j\|^2}$, where γ>0, was used in this study.

Each model is characterized by the parameters C and γ. As a means to determine a good parameter set, an estimate of the prediction accuracy is evaluated on a grid composed of (C,γ) pairs [4], where the pair leading to the best accuracy is chosen. Prediction accuracy is estimated through cross validation. This entails dividing the training data set into five subsets of equal size, where prediction is then performed over each subset using the classifier trained on the remaining subsets. The estimated accuracy is given by the percentage of correctly classified data instances. The best accuracy achieved for the classification of virus directed motion patterns was ~98%, corresponding to the parameters (C,γ) = ($3.6e^4$,33.1).

In summary, each trajectory is windowed, the obtained disjoint segments are mapped into low dimensional feature vectors [2] and classified as directed motion or not, based on the training data set.

The translation from 2D to 1D yields a direct correlation between distance traveled along microtubules and time. Consequently, some of the features defined in [2], which characterize the segment structure, are redundant. The same segmentation accuracy can be achieved through a decreased set of features, which results in a reduction of the computational complexity and a more robust classifier. In this work, the net squared distance and sum of squared step length were used:

$$\psi_1 = \left(\mathbf{x}_{end} - \mathbf{x}_{start}\right)^2, \qquad (4)$$

$$\psi_2 = \sum_i \left(\mathbf{x}_{i+1} - \mathbf{x}_i\right)^2. \qquad (5)$$

Dynein and Kinesin Dynamics. The virus particle is modeled as cargo that is moved

along a 1-dimensional microtubule by motor proteins. The movement of the virus is dictated by the number of bound motor proteins, and it is assumed that dynein (D) and kinesin (K) pull the virus in different directions. The maximum number of motor proteins that can affect the position of the virus is limited by the number of binding sites (ρ) on the viral capsid. The dynamics of the model are governed by the stochastic binding and unbinding of the dynein and kinesin particles and their stepping along the microtubule. The step sizes of the motor proteins, denoted by μ_D and μ_K are assumed to be constant (-8 nm for dynein, +8 nm for kinesin), and the displacement of the virus directly corresponds to one of the motor proteins taking a step in its characteristic direction.

Common Binding Sites, Model I. Comprises common binding sites for dynein and kinesin, and has been discussed in detail in the main text.

Separate Binding Sites, Model II. A second model is considered where the dynein and kinesin motor proteins have their own distinct binding sites on the virus (Fig. S1A). This effectively leads to a situation where there are ρ_D number of binding sites available for dynein and ρ_K for kinesin:

$$D \xrightarrow{d_\mu} D + \mu_D \qquad K \xrightarrow{k_\mu} K + \mu_K \qquad (6)$$
$$\rho_D \xrightarrow{d_\alpha} D \qquad \rho_K \xrightarrow{k_\alpha} K \qquad (7)$$
$$D \xrightarrow{d_\delta} \rho_D \qquad K \xrightarrow{k_\delta} \rho_K \qquad (8)$$

Single Motor Protein, Model III. A model involving only a single motor protein is considered, where dynein is able to move both to and from the cell periphery (Fig. S1B). The absence of kinesin reduces the number of events and parameters in the system, which is represented by:

$$D \xrightarrow{d_+} D + \mu_D \qquad D \xrightarrow{d_-} D - \mu_D \qquad (9)$$
$$\rho \xrightarrow{d_\alpha} D \qquad D \xrightarrow{d_\delta} \rho \qquad (10)$$

This model does not lead to directional runs, but rather the particle diffuses randomly.

Kinesin Binding to Dynein, Model IV. The last model presents a binding dependence between dynein and kinesin. The two families of motors maintain different motilities and preferential directions, but only dynein is allowed to attach to a virus particle (Fig. S1C). Kinesin can only bind indirectly to the viral cargo through the dynein complex. This implies that the number of binding sites available for kinesin (ρ_K) is, at most, equal to the number of currently attached dynein complexes. Furthermore the unbinding of a dynein particle can also cause the detachment of a bound kinesin. This model was used to investigate the dependence of the kinesin motor protein on dynein.

$$D \xrightarrow{d_\mu} D + \mu_D \qquad K \xrightarrow{k_\mu} K + \mu_K \qquad (11)$$
$$\rho_D \xrightarrow{d_\alpha} D \qquad \rho_K \xrightarrow{k_\alpha} K \qquad (12)$$

$$D \xrightarrow{d_\delta\left(1-\frac{K}{D}\right)} \rho_D \qquad D+K \xrightarrow{d_\delta\left(\frac{K}{D}\right)} \rho_D \qquad (13)$$

$$K \xrightarrow{k_\delta} \rho_K \qquad (14)$$

One possible mechanism of regulating motor cargo binding is that a motor binds indirectly to the cargo via another motor protein. In this model, kinesin is not able to bind to the virus particle directly, but rather binds to dynein. This model only exhibits minus-end directed motions, and is thus not able to account for the virus motility. This behavior can be explained by the inability of kinesin to bind the cargo independently of dynein. Therefore an unbalanced configuration in which kinesin is predominant can never occur. We conclude that model III and IV would therefore require further levels of regulation to exhibit bidirectional behavior.

Stochastic Simulation Algorithm. The Stochastic Simulation Algorithm (SSA) [5,6] simulates the time evolution of stochastic processes occurring at some prescribed rate. At each iteration of the SSA the process is advanced by a time unit depending on the overall propensity of the system and by an event that is selected depending on its relative propensity. The so-called propensity of an event is an unscaled probability for the event to occur in the interval $[t,t+\tau)$, where t is the current time and τ is the time-step. The propensities for events of the form

$$X_i \xrightarrow{k_i} X_j \qquad (15)$$

are calculated as $a_i = X_i k_i$, where i is the index of the event, X_i is the number of particles (e.g. this can be the number of molecules of a particular species if the events are chemical reactions or dynein proteins in the aforementioned models), and k_i is the rate of the process. Given that there are a total of M events involving N kinds of particles, the simulation is performed by executing three steps: 1) calculating the time-step, which is sampled from an exponential distribution:

$$\tau \sim \varepsilon\left(\frac{1}{\sum_{m=1}^{M} a_m}\right) \qquad (16)$$

then, 2) choosing the event that occurs in the interval [t,t+τ) by sampling from the point-wise probabilities $a_j / \sum_{m=1}^{M} a_m$, namely

$$P(j=l) = \frac{a_l}{\sum_{m=1}^{M} a_m} \qquad l = 1,\dots,M \qquad (17)$$

and lastly, 3) re-computing the propensities and updating the values of X_i for $i = 1,\dots,N$. These three steps are performed iteratively until the desired final time is reached.

Optimization of Parameters. The parameters in the models (e.g. for model I: d_μ, k_μ, d_α, k_α, d_δ, k_δ) are not specified a priori, but determined by the stochastic optimization algorithm called Covariance Matrix Adaptation Evolutionary Strategy (CMA-ES) [7,8]. This optimization technique has been proven to be effective in handling noise, multi-modality, and discontinuities in the cost function and it is well suited to the optimization of stochastic models.

The cost function is chosen as the sum of the symmetric Kullback-Leibler divergence (D_{SKL}) of directed motion length and velocity probability density functions (PDFs)

$$F = D_{SKL}^{length} + D_{SKL}^{velocity} \qquad (19)$$

Given a target PDF p(x) and a test PDF q(x), the D_{SKL} is defined as

$$D_{SKL} = \int_{-\infty}^{\infty} p(x) \log \frac{p(x)}{q(x)} dx + \int_{-\infty}^{\infty} q(x) \log \frac{q(x)}{p(x)} dx \qquad (18)$$

Here *p(x)* are the biological directed motion length and velocity distributions, while *q(x)* are the *in silico* distributions that are obtained for a given set of parameters. For each set of parameters to be evaluated, 3000 trajectories were stochastically simulated and segmented.

Sensitivity Analysis. Each of the six optimal parameters for the non-competing binding sites model with 14 receptors was perturbed keeping the other parameters fixed. This is equivalent to a crosscut of the cost function with respect to the perturbed parameter. The resulting values of the cost function are shown in Figure S3. The parabolic shapes of the perturbations indicate that a minimum was found by CMA-ES, and that local perturbations of the optimal parameters yield a less optimal set of parameters. In some cases, the optimal parameter found by CMA-ES is not the minimum in the crosscut (e.g. the stepping rate of kinesin in Fig S3), the reasons being that the model is inherently noisy and nonlinear.

Parallelization and computational cost. The optimization algorithm was implemented to allow parallel cost function evaluations according to both shared and distributed parallelization paradigms. More then 10^5 CPU hours were needed at the Swiss National Supercomputing Centre (CSCS) to perform the computations.

Bibliography

1. Sbalzarini I F, Koumoutsakos P (2005) Feature point tracking and trajectory analysis for video imaging in cell biology. Journal of Structural Biology 151:182-195.

2. Helmuth J A, Burckhardt C J, Koumoutsakos P, Greber U F, Sbalzarini I F (2007) A novel supervised trajectory segmentation algorithm identifies distinct types of human adenovirus motion in host cells. Journal of Structural Biology 159:347-358.

3. Chang C C, Lin C J (2001) LIBSVM: a library for support vector machines.

4. Hsu C W, Chang C C, Lin C J (2008) A practical guide to support vector classification.

5. Bortz A B, Kalos M H, Lebowitz J L (1975) New algorithm for Monte-Carlo simulation of Ising spin systems. Journal of Computational Physics 17:10-18.

6. Gillespie D T (1977) Exact stochastic simulation of coupled chemical reactions. J. Phys. Chem. 81:2340-2361.

7. Hansen N, Kern S (2004) Evaluating the CMA evolution strategy on multimodal test functions. Parallel Problem Solving from Nature - PPSN VIII 3242:282-291.

8. Hansen N, Ostermeier A (2001) Completely derandomized self-adaptation in evolution strategies. Evolutionary Computation 9:159-195.

9. King S J, Schroer T A (2000) Dynactin increases the processivity of the cytoplasmic dynein motor. Nature Cell Biology 2:20-24.

10. Berezuk M A, Schroer T A (2007) Dynactin enhances the processivity of kinesin-2. Traffic 8:124-129.

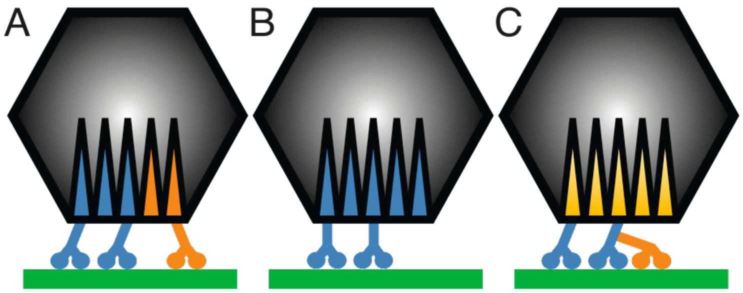

Fig. S1: Alternative models for motor-mediated virus movements on microtubules

A) In addition to model I (Fig. 2A), model II provides distinct dynein and kinesin binding sites on the virus particle allowing a tug-of-war between opposite polarity dynein (blue) and kinesin (orange) motors on a microtubule (green). B) Model III proposes unique binding sites, as shown for dynein (blue) allowing the protein to move in both directions. C) Model IV describes unique binding sites for dynein, to which kinesin is allowed to bind subsequently.

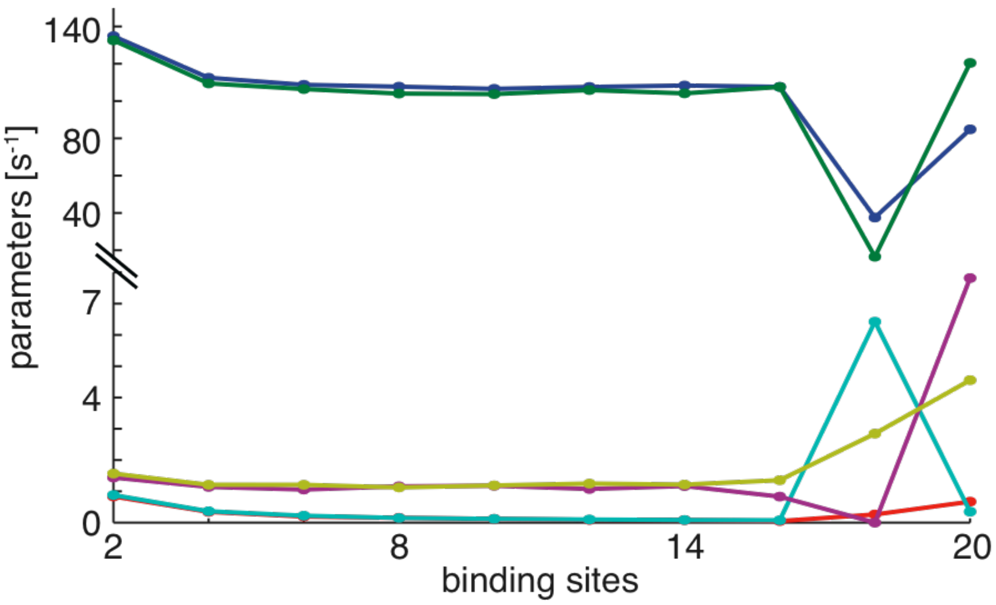

Fig. S2: Parameter values vs number of binding sites
Optimal parameter values (blue=d_μ, green=k_μ, red=d_α, cyan=k_α, magenta=d_δ, yellow=k_δ) obtained varying the number of motor binding sites for the overlapping binding sites model. As can be seen between 6 and 16 number of motor binding sites parameters are almost constant.

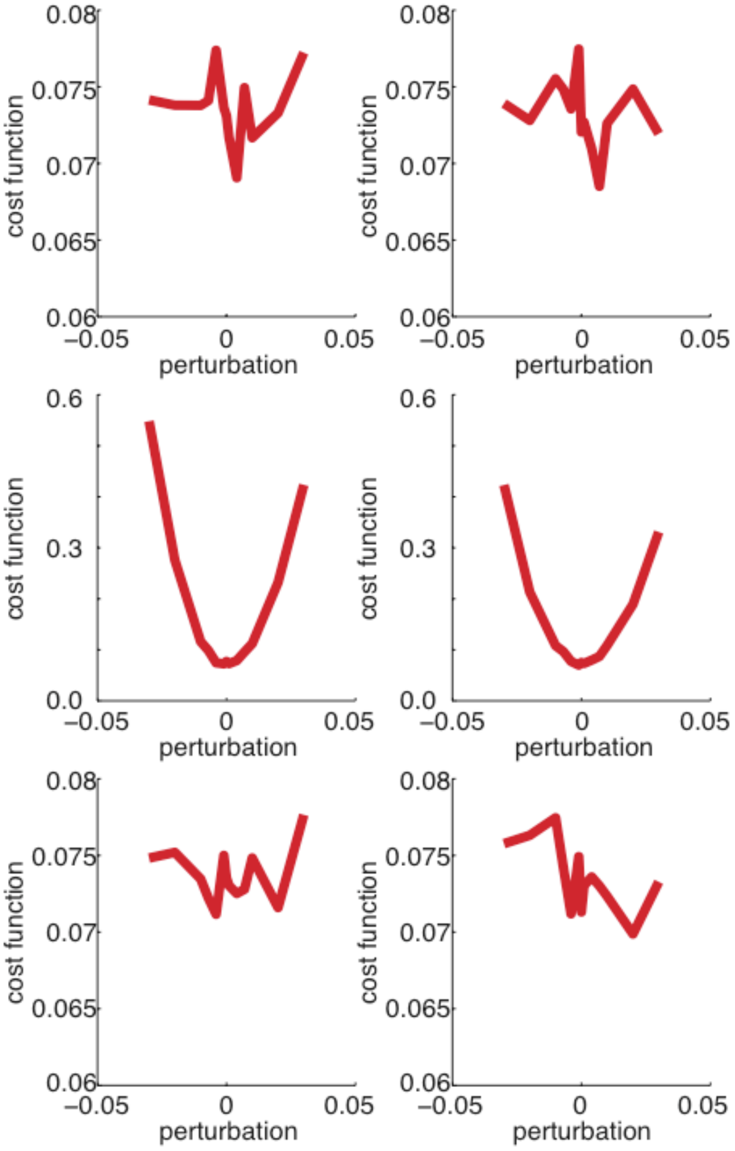

Fig. S3: Sensitivity Analysis.
The six optimal parameters for the non-competing binding sites model with 14 receptors were each perturbed by a quantity in the range between ± 0.05. Shown are (top) the stepping rates, (middle) binding rates, and (bottom) unbinding rates for dynein (left) and kinesin (right).

Binding sites	Parameters						
	d_μ	k_μ	d_α	k_α	d_δ	k_δ	F
2	134.70	132.56	0.8360	0.8815	1.4404	1.5681	0.553
4	112.51	109.45	0.3439	0.3634	1.1428	1.2099	0.141
6	108.72	106.42	0.1938	0.2215	1.0571	1.2010	0.088
8	107.72	104.01	0.1542	0.1496	1.1607	1.1252	0.075
10	106.56	103.73	0.1200	0.1215	1.1790	1.1896	0.070
12	107.59	105.89	0.0867	0.0993	1.0762	1.2479	0.069
14	108.33	104.13	0.0799	0.0843	1.1702	1.2162	0.065
16	107.62	107.57	0.0503	0.0760	0.8324	1.3570	0.083
18	37.54	16.51	0.2615	6.4234	0.0001	2.8456	2.156
20	84.83	120.44	0.6701	0.3506	7.8185	4.5548	3.612

Table S1:
Optimized cost function and parameter values against number of motor binding sites for the overlapping binding sites model. Different optimization runs were performed varying the number of motor binding sites. Every entry in the table shows the best set of parameters obtained. d_μ and k_μ correspond to the dynein and kinesin stepping rate, respectively, and are expressed in stepping events per second. The single motor speed is given by d_μ or k_μ times the step size (8nm). d_α and k_α are the binding rates for dynein and kinesin. They are expressed in events per second per receptor. d_δ and k_δ represent the unbinding rates for dynein and kinesin and they are expressed as detachment events per second. F is the cost function value at the end of the optimization.
